# Early establishment of chloride homeostasis in CRH neurons is altered by prenatal stress leading to fetal HPA axis dysregulation

**DOI:** 10.3389/fnmol.2024.1373337

**Published:** 2024-03-21

**Authors:** Miho Watanabe, Adya Saran Sinha, Yohei Shinmyo, Atsuo Fukuda

**Affiliations:** Department of Neurophysiology, Hamamatsu University School of Medicine, Hamamatsu, Japan

**Keywords:** HPA axis, CRH neuron, GABA, chloride homeostasis, KCC2, prenatal stress

## Abstract

Corticotropin-releasing hormone (CRH) neurons play an important role in the regulation of neuroendocrine responses to stress. The excitability of CRH neurons is regulated by inhibitory GABAergic inputs. However, it is unclear when GABAergic regulation of CRH neurons is established during fetal brain development. Furthermore, the exact progression of the developmental shift of GABA action from depolarization to hyperpolarization remains unelucidated. Considering the importance of CRH neuron function in subsequent hypothalamic-pituitary-adrenal (HPA) axis regulation during this critical phase of development, we investigated the ontogeny of GABAergic inputs to CRH neurons and consequent development of chloride homeostasis. Both CRH neuron soma in the paraventricular nucleus (PVN) and axons projecting to the median eminence could be identified at embryonic day 15 (E15). Using acute slices containing the PVN of CRF-VenusΔNeo mice, gramicidin perforated-patch clamp-recordings of CRH neurons at E15, postnatal day 0 (P0), and P7 were performed to evaluate the developmental shift of GABA action. The equilibrium potential of GABA (E_GABA_) was similar between E15 and P0 and showed a further hyperpolarizing shift between P0 and P7 that was comparable to E_GABA_ values in adult CRH neurons. GABA primarily acted as an inhibitory signal at E15 and KCC2 expression was detected in CRH neurons at this age. Activation of the HPA axis has been proposed as the primary mechanism through which prenatal maternal stress shapes fetal development and subsequent long-term disease risk. We therefore examined the impact of maternal food restriction stress on the development of chloride homeostasis in CRH neurons. We observed a depolarization shift of E_GABA_ in CRH neurons of pups exposed to maternal food restriction stress. These results suggest that Cl^–^ homeostasis in early developmental CRH neurons attains mature intracellular Cl^–^ levels, GABA acts primarily as inhibitory, and CRH neurons mature and function early compared with neurons in other brain regions, such as the cortex and hippocampus. Maternal food restriction stress alters chloride homeostasis in CRH neurons of pups, reducing their inhibitory control by GABA. This may contribute to increased CRH neuron activity and cause activation of the HPA axis in pups.

## Introduction

The hypothalamic-pituitary-adrenal (HPA) axis is a key physiological element in how an organism responds to challenges manifested by either internal or external stressors at any given phase of life (Charmandari et al., 2005). Exposure to a variety of stress leads to activation of corticotropin-releasing hormone (CRH) neurons in the hypothalamic paraventricular nucleus (PVN), which release CRH into portal circulation. This leads to subsequent release of adrenocorticotropic hormone from the anterior pituitary into systemic circulation, culminating in activation of the adrenal cortex, which stimulates synthesis and secretion of cortisol in humans and corticosterone in rodents into the systemic circulation (Ulrich-Lai and Herman, 2009). Thereafter, these glucocorticoids initiate multiple cascades that both limit non-essential energy expenditure and increase energy production to cope with these stressors.

For these reasons, this population of CRH neurons have been the subject of extensive studies investigating their normal physiology and plasticity during stress (Herman and Tasker, 2016). Release of the major inhibitory neurotransmitter γ-aminobutyric acid (GABA) onto CRH neurons from multiple afferent inputs originating in diverse regions of the brain is a key regulator of their activity (Levy and Tasker, 2012). More recent evidence also indicates a GABA-mediated novel mechanism of steady-state CRH release from CRH neuron axon terminals in the median eminence (ME) (Kakizawa et al., 2016; Yesmin et al., 2022). Together, these findings highlight the importance of GABAergic signaling in CRH neuron activity and downstream HPA axis regulation.

The inhibitory action of GABA is closely linked to the phenomenon of chloride homeostasis wherein, following development, low intracellular Cl^–^ concentrations [Cl^–^]_i_ are maintained by optimal functioning of the K^+^-Cl^–^ cotransporter KCC2 in mature neurons (Rivera et al., 1999). However, early in development, relatively higher [Cl^–^]_i_ due to suboptimal KCC2 function results in depolarizing GABA action (Ben-Ari, 2002). As KCC2 function develops over time during brain development, the shift from depolarizing to hyperpolarizing GABA action is enacted in most brain regions during the second postnatal week (Watanabe and Fukuda, 2015). Developmental studies examining the formation of hypothalamic nuclei indicate that the birthdate of CRH neurons reportedly peaks around E13 in the rat and mouse brain (Keegan et al., 1994; Markakis and Swanson, 1997), with distinct identifiable structures organizing as early E14–E15 in the PVN. However, what is not addressed is whether the fetal HPA axis becomes functional at this early stage of development. In addition, ontogenesis of CRH neuron-specific KCC2 function regulation of chloride homeostasis, and establishment of mature GABAergic control of the HPA axis remain to be investigated.

The significance of this developmental phase can be further assessed based on a multitude of evidence from both retrospective human studies and experimental prenatal stress animal models (Weinstock, 2008). These reports indicate frequent microscopic and macroscopic alterations in brain structures that culminate in behavioral abnormalities ranging from attention and learning deficits to generalized anxiety and depression in offspring. The dysregulation of both maternal and fetal HPA axes has been proposed as a likely underlying mechanism (Charil et al., 2010). Observations indicating dynamic regulation of KCC2, disruption of [Cl^–^]_i_, and reverting back to depolarizing GABA action in conditions such as traumatic brain injury, epilepsies, and spinal cord injuries emphasizes their role in the etiology of neuropathologies (Kaila et al., 2014; Fukuda and Watanabe, 2019). In adult rodents, when GABAergic inhibition in CRH neurons is well established, exposure to chronic stress has been shown to affect the plasticity of GABAergic synapses resulting in HPA axis dysregulation (Maguire, 2014). Notably, in response to episodes of stress, GABA action switches from inhibitory to excitatory as a result of KCC2 downregulation (Hewitt et al., 2009; Sarkar et al., 2011). Therefore, the impact of stress on excitability of CRH neurons and overall physiological functioning of the HPA axis is critically dependent on the interplay between KCC2 function and GABA action. It is therefore conceivable to suggest that perinatal stress may affect KCC2 function, the development of GABAergic inhibition onto CRH neurons, and HPA axis regulatory mechanisms in progeny.

In view of this possibility, we here investigated the ontogeny of GABAergic inputs to CRH neurons and consequent development of chloride homeostasis. Furthermore, we examine the impact of perinatal stress in this regulatory sequence.

## Materials and methods

### Ethical approval

All experimental procedures were in accordance with guidelines issued by the Hamamatsu University School of Medicine on the ethical use of animals for experimentation and approved by the Committee for Animal Care and Use (No. 23-049). All efforts were made to minimize the number of animals used and their suffering.

### Animals

The CRF-VenusΔNeo mouse was produced as described previously (Nagai et al., 2002; Itoi et al., 2014; Kono et al., 2017) and kindly gifted from RIKEN BioResource Research Center (BRC No. RBRC09893). CRF-VenusΔNeo mice express Venus under control of the *CRF* (CRH) promoter. These mice were bred on a C57BL/6J background. Genotyping of CRF-VenusΔNeo mice was carried out by PCR of mouse tail DNA using the following primers: 5’-AGGACGACGGCAACTACAAG-3’ and 5’-TCTCGTTGGGGTCTTTGCTC-3’. In the present study, male CRF-VenusΔNeo mice were placed with female C57BL/6J mice (CLEA Japan, Tokyo, Japan) overnight in a cage. The day when a vaginal plug was identified was defined as embryonic day (E) 0 and gestational day (G) 0 for the mother. Mice were housed with a 12-h light/dark cycle (lights on at 07:00 am) and access to food and water *ad libitum* unless otherwise noted.

### Acute brain slice preparation

Coronal brain slices (300 μm in thickness) including the hypothalamus were obtained from male and female mice at E15–E17 and postnatal days (P) 0–2 and 7–9. Under deep anesthesia with ketamine (150 mg/kg body weight)/xylazine (30 mg/kg body weight), mice were decapitated, and their brains were removed. Slices were made using a Vibrating Microtome 7000 (Campden Instruments, Loughborough, Leicestershire, UK) in an ice-cold oxygenated sucrose solution containing (in mM) 220 sucrose, 120 NaCl, 2.5 KCl, 0.5 CaCl_2_, 1.25 NaH_2_PO_4_, 1 MgCl_2_, 26 NaHCO_3_, 30 glucose, and 10 MgSO_4_ (pH 7.4). Slices were maintained in standard artificial cerebrospinal fluid (ACSF) consisting of (in mM) 126 NaCl, 2.5 KCl, 1.25 NaH_2_PO_4_, 2 MgSO_4_, 2 CaCl_2_, 26 NaHCO_3_, and 20 glucose (pH 7.4) equilibrated with 95% O_2_ and 5% CO_2_ at room temperature before recording. For recording, slices were transferred to a recording chamber that was perfused with oxygenated ACSF.

### Electrophysiology

Electrophysiological recordings were performed using a MultiClamp 700B amplifier (Molecular Devices, San Jose, CA, USA) and pClamp10 software (Molecular Devices, San Jose, CA, USA). Currents were filtered at 2 kHz and digitized at 10 kHz using DigiData1440A. Data were analyzed offline using Clampfit10 (Molecular Devices, San Jose, CA, USA). To estimate the reversal potential of GABA (E_GABA_) of CRH neurons in hypothalamic slices, we performed gramicidin-perforated patch-clamp recording to acquire GABA-evoked responses with native intracellular Cl^–^ concentrations. CRH neurons expressing Venus were selected under epifluorescent illumination. Patch electrode pipettes (4 to 6 megaOhms) were pulled from borosilicate glass capillaries on a P-97 puller (Sutter Instrument, Novato, CA, USA) and filled with pipette solution composed of 150 mM KCl and 10 mM HEPES (pH 7.2) supplemented with gramicidin (Sigma-Aldrich, St. Louis, MO, USA). Gramicidin was dissolved in methanol to prepare a stock solution of 10 mg/mL and then diluted in pipette solution to a final concentration of 30 μg/mL. Recordings were made in ACSF supplemented with 10 μM CNQX (Sigma-Aldrich, St. Louis, MO, USA), 40 μM D-AP5 (Cayman Chemical, Ann Arbor, MI, USA), 3 μM CGP55845 (Sigma-Aldrich, St. Louis, MO, USA), and 500 nM tetrodotoxin (WAKO, Osaka, Japan) to block AMPA- and NMDA-type glutamate receptors, GABA_B_ receptors, and voltage-gated Na^+^ channels, respectively. The reversal potential of 100 μM GABA-induced current was measured at −70 mV holding potential (*V*_H_), and 1-sec voltage ramps from −90 to −10 mV applied before and during GABA application. E_GABA_ was estimated by measuring the voltage at which the *I*-*V* relationships before and during GABA application intersected (Saitsu et al., 2016). Series resistance (R_s_) was compensated by 70%. Membrane potential values were corrected for the voltage drop across the uncompensated R_s_ using the following equation: *V*_corr_ = *V*_com_ – *I*_clamp_ × R_s_, where *V*_com_ is the command potential and *I*_clamp_ is the clamp current. To determine *E*_GABA_, these values were plotted as a function of the R_s_-corrected membrane potential. GABA puffs (5-sec duration) were applied through a patch pipette approximated to the soma using an IM-300 Programmable Microinjector (Narishige, Tokyo, Japan). To study the effect of GABA on membrane potential, current-clamp recording was performed on CRH neurons of P0-P2 mice in gramicidin-perforated patch mode. The bridge balance circuit was applied while recording voltage changes. Recordings were made in ACSF supplemented with 10 μM CNQX, 40 μM D-AP5, and 3 μM CGP55845. GABA puffs (3-sec duration) were applied through a patch pipette approximated to the soma and GABA-induced potential was recorded. Resting membrane potential (RMP) was calculated from traces at zero current injection (*I* = 0) levels. The calculated liquid junction potential of −3.6 mV was not corrected because the higher K^+^ ion concentration in the electrode solution compared with the cytosol would be negated by the E_K_ of approximately +4 mV (Kim and Trussell, 2007).

### Immunohistochemistry

Under deep anesthesia with ketamine/xylazine, E15 C57BL/6J mice were transcardially perfused with 4% paraformaldehyde (PFA) in 0.1 M phosphate buffer. Mouse brains were rapidly removed and post fixed for 2 h in 4% PFA at 4°C, followed by 20 and 30% sucrose in phosphate-buffered saline (PBS) at 4°C overnight. Brains were sectioned coronally (30 μm) with a cryostat (HM560R; Zeiss Microm). Subsequently, sections were incubated for 1 h in blocking solution [10% normal goat serum in 0.1% Tween 20 (Sigma-Aldrich, St. Louis, MO, USA) in PBS (PBS-T)] at room temperature before incubation for 48 h at 4°C with guinea pig anti-CRH (1:800; Peninsula Laboratories, San Carlos, CA, USA) and rabbit anti-KCC2 (1:500; Merck Millipore, Burlington, MA, USA) diluted in PBS-T. Subsequently, sections were washed several times with PBS-T and incubated with the following secondary antibodies: Alexa Fluor 488-conjugated goat anti-guinea pig and Alexa Fluor 594-conjugated goat anti-rabbit (1:1,000; Molecular Probes, Eugene, OR, USA). After several washes with PBS-T, the slides were mounted with PermaFluor aqueous mounting medium (Thermo Fisher Scientific, Wilmington, DE, USA) and coverslipped. Slices were imaged using a confocal laser-scanning microscope (FV1000-D, Olympus, Tokyo, Japan).

### Food restriction stress

CRF-VenusΔNeo pregnant mice were subjected to 70% food restriction stress from G10 to G18. For the food restriction experiment, daily food consumption of pregnant mice was measured from G10 to G18 and average food consumption per gram of body weight was calculated. Pregnant mice were given a food pellet equal to 70% of their average daily food intake from G10 to G18. Body weights were measured during 70% food restriction. For hormone assays, pregnant mice and fetuses at E18 were used. For electrophysiology experiments, pups were raised by naive surrogate mothers with the same delivery date at P0 and recordings were performed using P0–P2 mice.

### Hormone assay

Pregnant mice at E18 were sacrificed by cervical dislocation. Mice were decapitated and the trunk blood was collected into polyethylene tubes containing EDTA-2K (Becton, Dickinson and Company, Franklin Lakes, NJ, USA). Samples were collected between 09:00 and 11:00 am Samples were centrifuged at 800 *g* for 20 min at 4°C, and the serum was collected and stored at −80°C until further use. As fetuses at E18 provide an insufficient amount of blood, the fetus body was homogenized with a homogenizer in 500 μL of 1 N HCl, followed by centrifugation at 9,100 *g* for 15 min at 4°C. The resulting supernatant was collected and stored at −80°C. For analysis, 100 μL of each supernatant was lyophilized and reconstituted in 300 μL of radioimmunoassay (RIA) buffer [0.1 M PB (pH 7.4) containing 0.05% NaN_3_ and 0.1% Triton X-100]. Corticosterone levels were determined by RIA, as previously reported (Yesmin et al., 2022). Briefly, a 25-μL sample of serum was boiled at 98°C for 5 min. Ice-cold RIA buffer was used for dilution and ^125^I-corticosterone (Institute of Isotopes) was used as the label. A mixture comprising 100 μL of corticosterone standard or sample, 100 μL of corticosterone antiserum, and 100 μL of ^125^I-labeled corticosterone was incubated for 24 h at 4°C. Antibody-bound and antibody-free corticosterone were separated via incubation with 100 μL of a secondary antibody (bovine γ-globulin) and 400 μL of 25% polyethylene glycol, followed by centrifugation at 800 *g* for 15 min at 4°C. The radioactivity of bound antibodies was counted using a γ-counter (ARC-7010, Aloka, Tokyo, Japan). The assay did not cross-react with other corticosteroids and its sensitivity was 2 pg/tube.

### Statistics

All statistical analyses were performed using GraphPad Prism (version 8; GraphPad Software). The data was evaluated for normal distribution based on Shapiro–Wilk test and measure of variance was based on F-Test. If the data was found to be normally distributed, we performed unpaired *t*-test. When the data failed tests for normality, nonparametric tests for comparison between two groups (Mann–Whitney *U* test) and multiple groups (Kruskal–Wallis one-way ANOVA followed by Dunn’s multiple comparison test) were used, respectively. Data are presented as mean ± standard error of the mean (SEM) as well as median in the text. When the data is statistically compared using nonparametric test, graphical representation of results in figures show box plot that indicates median, 25 and 75th percentile, and whiskers extending from each end of a box depict the minimum and maximum ranges. The individual data points are shown with dots. The confidence interval for statistics was set at 95% for all data comparisons. *P*-value < 0.05 is indicated as * and < 0.01 as **. No difference between groups was shown as ns.

## Results

### E_GABA_ of CRH neurons during developmental period

**FIGURE 1:**
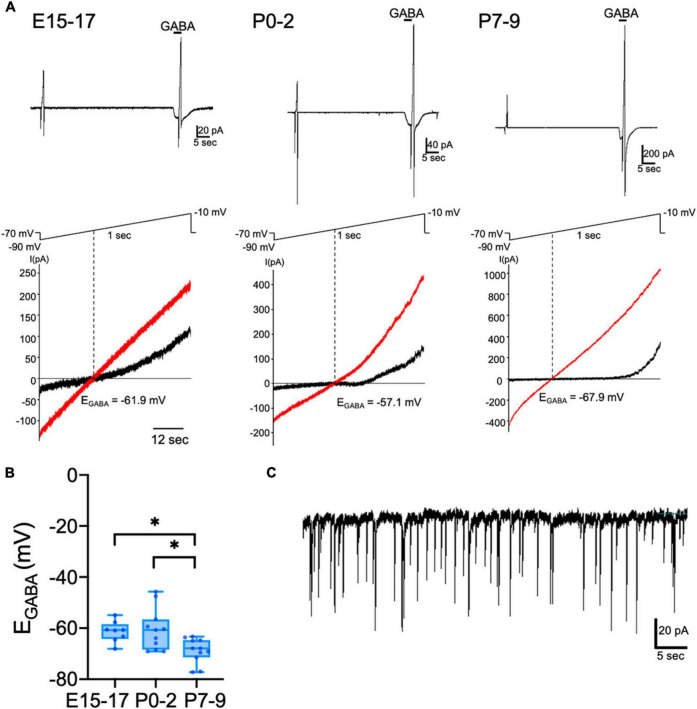
Developmental change of E_GABA_ in CRH neurons. Gramicidin-perforated patch-clamp recordings of CRH neurons from E15–E17, P0–P2, and P7–P9 mice. **(A)** A representative 100 μM GABA-evoked current trace at –70 mV holding potential (upper panel). The duration of GABA application is indicated with horizontal bars. Two 1-sec voltage ramps from –90 to –10 mV were applied before and during 5-sec puff application of GABA; sample *I*-*V* curves before (black) and after GABA application (red) (lower panel). E_GABA_ was estimated from the voltage axis intercept (detailed further in “Materials and methods”). **(B)** E_GABA_ of CRH neurons at E15–E17, P0–P2, and P7–P9 (*p < 0.05 by Kruskal–Wallis test; E15–E17: *n* = 8, P0–P2: *n* = 11, P7–P9: *n* = 11; closed circle indicates single cells). The lines in each box depict the lower quartile, median, and upper quartile values. The whiskers extending from each end of a box depict the minimum and maximum ranges. **(C)** sIPSCs recorded from E15 CRH neurons after the neuronal membrane was ruptured during perforated-patch clamp-recording. Scale bar as shown in the figure inset.

The activity of CRH neurons is reportedly regulated by GABA in adult mice (Camille Melón and Maguire, 2016). GABAergic inputs exert inhibitory action on the somata of CRH neurons through activation of GABA_A_ receptors (GABA_A_Rs) (Kakizawa et al., 2016). To investigate when GABAergic regulation of CRH neurons is established and the development of chloride homeostasis, we measured E_GABA_ determined primarily by the Cl^–^ equilibrium potential [reflecting intracellular [Cl^–^]_i_ (Medina et al., 2014)] using gramicidin-perforated patch-clamp technique, which avoids disruption of native neuronal [Cl^–^]_i_. Using CRF-VenusΔNeo mice, CRH neurons were identified by Venus fluorescence. We recorded E_GABA_ of CRH neurons in hypothalamic slices from E15–E17, P0–P2, and P7–P9 mice (Figure 1A). The mean E_GABA_ of CRH neurons was −61.3 ± 1.4 mV at E15–E17 and −60.6 ± 2.4 mV at P0–P2 (Figure 1B). We observed no significant differences in mean E_GABA_ values between E15–E17 and P0–P2. The E_GABA_ of P7–P9 CRH neurons was significantly shifted to more hyperpolarized values (P7–P9: −68.8 ± 1.5 mV) compared to E15–E17 and P0–P2 CRH neurons (*p* < 0.05 by Kruskal–Wallis test). The corresponding median values for E15-E17 and P0-P2 were −60.8 and −60.7 mV, respectively, whereas for P7-P9 was −67.9 mV. These results suggest that the developmental shift of E_GABA_ occurs between P0 and P7, and E_GABA_ of perinatal CRH neurons is hyperpolarized compared to neurons in other brain regions, such as the cortex and hippocampus. During gramicidin-perforated patch-clamp recording, the neuronal membrane occasionally ruptured and spontaneous inhibitory postsynaptic currents (sIPSCs) were observed from E15 CRH neurons when voltage-clamped (V_H_) at −70 mV (Figure 1C), suggesting that E15 CRH neurons express synaptic GABA_A_Rs and receive GABAergic inputs at E15.

### GABAergic inhibitory regulation of CRH neurons is established during early development

**FIGURE 2:**
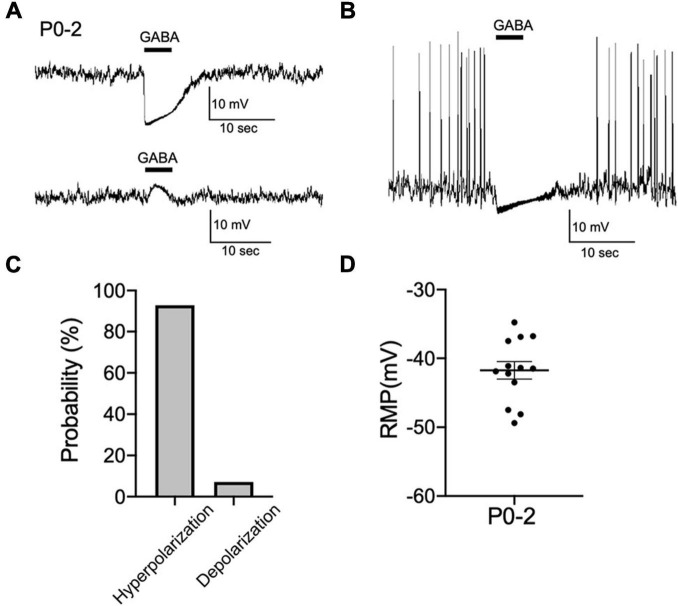
GABAergic inhibitory regulation of CRH neurons is established during early development. **(A)** Representative current-clamp recordings from P0–P2 CRH neurons in gramicidin-perforated patch mode showing the effect of 100 μM GABA on membrane potential. Five-second application of GABA hyperpolarized (upper panel) or depolarized (lower panel) membrane potential. **(B)** Application of GABA blocked spontaneous action potentials in P0–P2 CRH neurons (*n* = 2). **(C)** Summary of the proportions of each response type (depolarization or hyperpolarization) recorded upon GABA application in P0–P2 CRH neurons (*n* = 14). **(D)** The RMP of CRH neurons at P0–P2 (*n* = 13). Error bars represent SEM. Scale bar as shown in the figure inset.

Whether activation of GABA_A_ receptors results in depolarization or hyperpolarization of membrane potential depends on the relationship of E_GABA_ to the RMP. Next, we performed current-clamp recordings from CRH neurons in hypothalamic slices obtained from P0 mice and examined the effect of GABA on membrane potential changes. Most CRH neurons showed hyperpolarizing responses to brief application of GABA (92%; 13 out of 14 neurons; Figures 2A, C). Only one CRH neuron displayed a depolarizing response to GABA (8%: 1 out of 14 neurons). Two CRH neurons showed spontaneous action potentials that were inhibited by GABA application (Figure 2B). The RMP of CRH neurons at P0–P2 was −41.7 ± 1.3 mV (Figure 2D) and positive to E_GABA_. These results suggest that GABA action is already inhibitory at P0–P2.

### CRH neuronal somata express KCC2 at E15

**FIGURE 3:**
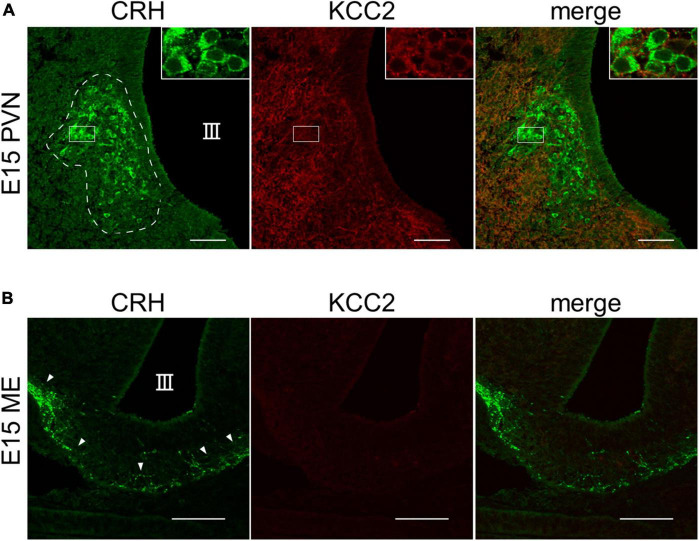
KCC2 is expressed in the somata of CRH neurons in PVN of E15–E17 mice. **(A)** Representative images of PVN from WT E15 mice immunostained for CRH (green) and KCC2 (red). III, third ventricle. White dotted line indicates the PVN with cell bodies of CRH neurons. **(B)** Representative images of ME from WT E15 mice immunostained for CRH (green) and KCC2 (red). III, third ventricle. White arrowheads indicate CRH neuron terminals in the ME. Scale bars: 50 μm.

The chloride cotransporter KCC2 is essential for GABAergic inhibition. In the cortex and hippocampus, the developmental shift of GABA action from excitatory to inhibitory, which results from KCC2 dependent Cl^–^ extrusion, occurs between P7 and P14 (Ben-Ari, 2002; Blaesse et al., 2009). Therefore, to examine if KCC2 is expressed in CRH neurons of E15 wild-type (WT) mice, we performed immunohistochemistry for CRH and KCC2. We observed CRH expression in the cell bodies of CRH neurons in the PVN and CRH terminals in the ME (Figures 3A, B), suggesting that CRH neurons already exist in the PVN and CRH axons project to the ME at E15. KCC2 expression in the somata of CRH neurons in the PVN was detected at E15, albeit weakly (Figure 3A), suggesting that it is expressed and functional at this age. In the ME, KCC2 expression in the CRH neuron terminals was not detected, similar to our report that KCC2 is not expressed in the CRH neuron terminals of adult mice (Figure 3B; Kakizawa et al., 2016). Cl^–^ homeostasis in early developmental CRH neurons attains mature [Cl^–^]_i_ levels with GABA acting in a predominantly inhibitory manner during their early development.

### Maternal food restriction stress causes a depolarizing shift in E_GABA_ of CRH neurons in pups

**FIGURE 4:**
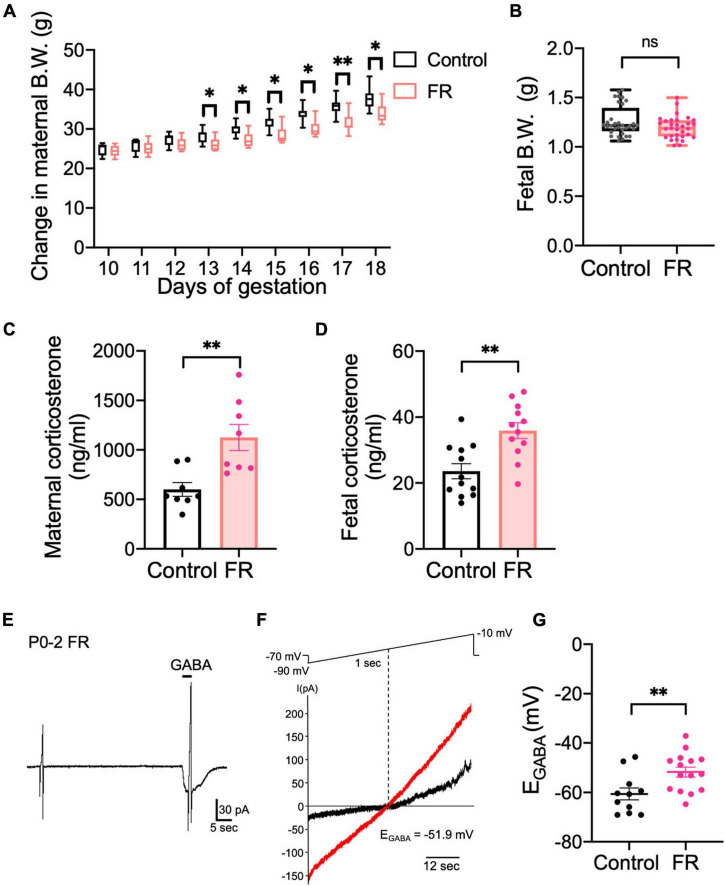
Maternal food restriction stress causes a depolarizing shift in E_GABA_ of CRH neurons in pups. **(A)** Body weights of control and FR mothers. The lines in each box depict the lower quartile, median, and upper quartile values. The whiskers extending from each end of a box depict the minimum and maximum ranges (*p < 0.05, **p < 0.01 by Mann–Whitney *U* test; *n* = 8 in each group). **(B)** Body weights of pups from control or FR mothers (ns, not significant; control: *n* = 12, FR: *n* = 12; closed circle indicates single cells). **(C)** Serum corticosterone level of control and FR mothers (**p < 0.01 by unpaired *t*-test, *n* = 8 in each group; closed circle indicates single cells). Error bars represent SEM. **(D)** Fetal corticosterone level of control and FR mothers (**p < 0.01 by unpaired *t*-test; control: *n* = 12, FR: *n* = 12; closed circle indicates single cells). Error bars represent SEM. **(E)** Representative 100 μM GABA-evoked current traces at –70 mV holding potential in CRH neurons of P0–P2 pups from FR mothers. Currents were recorded under gramicidin-perforated voltage-clamp condition. The duration of GABA application is indicated with horizontal bars. **(F)** Two 1-sec voltage ramps from –90 to –10 mV were applied before and during 5-sec puff application of 100 μM GABA; sample *I*-*V* curves before (black) and after GABA application (red). E_GABA_ was estimated from the voltage axis intercept (detailed further in “Materials and methods”). **(G)** Plot of E_GABA_ of CRH neurons from pups of control or FR mothers (**p < 0.01 by unpaired *t*-test; control: *n* = 11, FR: *n* = 15; closed circle indicates single cells). Error bars represent SEM.

Stress reportedly inhibits KCC2 function in adult CRH neurons of the PVN (Hewitt et al., 2009; Sarkar et al., 2011). Therefore, we examined whether prenatal stress affects chloride homeostasis in CRH neurons during early development. We used a maternal food restriction stress model that subjected pregnant mice to 70% food restriction (FR) from G10 to G18. Prenatal stress began on G10 because it corresponds with development of the fetal central nervous system (Clancy et al., 2001) and minimizes premature termination of the pregnancy as a result of excessive stress. First, we confirmed that FR affects the body weights and corticosterone levels of both the mother and fetus. Body weights of FR mothers were significantly decreased from G13 to G18 compared with control mothers (Figure 4A; *p* < 0.05, *p* < 0.01 by Mann–Whitney *U* test; median value: Control: 28.9 g and FR: 25.8 g at E13, Control: 35.85 g and FR: 30.6 g at E17). Fetal body weights from FR mothers tended to decrease, although not significantly (Figure 4B; ns by Mann–Whitney *U* test; Control: 1.261 ± 0.03 g; median value: 1.217 g, FR: 1.196 ± 0.02 g; median value: 1.19 g). Maternal serum corticosterone levels were significantly increased in FR mothers compared with control mothers (Figure 4C; *p* < 0.01 by unpaired *t*-test). Pups from FR mothers showed increased corticosterone levels compared with control pups (Figure 4D; *p* < 0.01 by unpaired *t*-test). These results confirm that FR activated the HPA axis of mothers and pups. To examine the effects of FR on chloride homeostasis in CRH neurons, we examined the effects of FR on E_GABA_ of CRH neurons from P0–P2 pups. E_GABA_ of CRH neurons from FR mothers was shifted to more depolarized values (Figures 4E–G; Control: −60.6 ± 2.4 mV, FR: −51.8 ± 2.0 mV; *p* < 0.01 by unpaired *t*-test) compared with previously observed E_GABA_ values from control pups (shown in Figure 1), suggesting that FR alters the chloride homeostasis of CRH neurons and reduces their inhibitory control by GABA. Such alterations may contribute to increased CRH neuron activity and cause activation of the HPA axis.

## Discussion

Our investigations examining the establishment of GABAergic innervation onto CRH neuron somata and sequela of the developmental shift of GABA action reveal that inhibitory GABA function is established by P0–P2 and undergoes a subsequent hyperpolarizing shift around P7–P9. This result may be facilitated by early KCC2 expression observed at E15 in CRH neurons. In addition, we observed spontaneous firing of CRH neurons at P0–P2 that could be inhibited by GABA application, indicating probable regulation of the fetal HPA axis by GABA function. Furthermore, application of food restriction stress during this critical phase of gestation produced a significant depolarizing shift of E_GABA_, suggesting food restriction stress alters chloride homeostasis.

The timing of Cl^–^ homeostasis maturation varies by brain region and cell type (Watanabe and Fukuda, 2015). Developmental decreases in [Cl^–^]_i_ occur in parallel with maturation of the nervous system. It occurs early in the spinal cord, followed by the hypothalamus and thalamus, and finally the limbic system and cortex. Neurons in the spinal cord and medulla originate mainly between E12–E13, while those in the thalamus, hypothalamus, and amygdala arise from E13–E16. In the neocortex, neurons originate between E16–E18; whereas in the hippocampus, pyramidal neurons arise between E17–E19 and dentate granule cells arise after birth (Wang et al., 2002). The sequence of KCC2 mRNA expression appears to follow the maturation sequence of neurons. KCC2 mRNA expression is detected in the spinal cord at E12, thalamus and hypothalamus at E14, and cortex and hippocampus at P15 (Hübner et al., 2001; Li et al., 2002; Shimizu-Okabe et al., 2002; Wang et al., 2002; Stein et al., 2004). In the hippocampus and cortex, the developmental shift of GABA from excitatory to inhibitory occurs during the second postnatal week (Kahle et al., 2008; Ben-Ari et al., 2012; Watanabe et al., 2019). In the present study, KCC2 was already observed in CRH neurons at E15. E_GABA_ showed hyperpolarized values that are comparable to E_GABA_ values in adult CRH neurons and GABA primarily acted as an inhibitory signal early in development, suggesting that CRH neurons mature and function earlier than neurons in the cortex and hippocampus. In addition, sIPSCs could be observed at E15, indicating the establishment of GABAergic synapses onto CRH neuron somata at an early phase of development. As previous reports also observed extrasynaptic GABA_A_R-mediated constraint of adult CRH neuron activity, it is probable that ambient GABA in the PVN exerts inhibitory control on these neurons early in development (Lee et al., 2014; Colmers and Bains, 2018). We also report a significant hyperpolarizing shift in E_GABA_ values at P7–P9 compared with E15–E17 and P0–P2. Interestingly, this phase coincides with the well-characterized stress hyporesponsive period (Vázquez, 1998). We hypothesize that the observed shift in the E_GABA_ value could be physiologically relevant in limiting the activation of CRH neurons during this period.

Some of the earliest electrophysiological evaluations of adult CRH neurons revealed deinactivation of a specific membrane conductance following hyperpolarization (Tasker and Dudek, 1991). This conductance facilitated generation of small low-threshold potentials in these neurons. Later, this voltage-dependent conductance was used as a distinguishing feature of parvocellular neurons (CRH, somatostatin neurons) in the PVN (Hoffman et al., 1991). More recently, with the advent of transgenic reporter mice, specific targeting of individual populations of cells in the PVN has been accomplished (Sarkar et al., 2011; Wamsteeker Cusulin et al., 2013). Applying a similar strategy for identifying CRH neurons in the developing hypothalamus (Kono et al., 2017), we evaluated the establishment of GABAergic inhibition onto these neurons and the ontogenesis of Cl^–^ homeostasis.

The development of neuronal membrane excitability of CRH neurons is critical for establishment of the HPA axis. In adult CRH neurons, the slope of frequencies of action potentials fired in response to current injection (*f-I* curve) displays linearity (Wamsteeker Cusulin et al., 2013). This active property of CRH neurons develops as early as P7 and the density of sodium and potassium channels is further enhanced with age (Melnick et al., 2007), suggesting that CRH neurons can modulate the HPA axis at an early phase. We observed spontaneous action potential firing as early as P0–P2, indicating functional sodium and potassium channels at this age. Therefore, it seems probable that CRH neurons are active early in development and the HPA axis could potentially be established at this stage. Furthermore, the early shift of GABA to hyperpolarizing function may parallel the early development of membrane excitability and act as a restraint for excitation of CRH neurons during the critical phase around birth.

We show that maternal food restriction stress shifted E_GABA_ to positive values in the CRH neurons of pups. Stress reportedly alters GABA signaling in adult CRH neurons and neurons in other brain regions. Acute restraint stress decreases the total and surface expression of KCC2, causing a depolarizing shift in E_GABA_ in CRH neurons of the PVN due to dephosphorylation of serine residue 940 (S940) (Hewitt et al., 2009; Sarkar et al., 2011). Chronic social defeat stress causes dephosphorylation at S940 and downregulation of KCC2 in the PVN, and increases plasma corticosterone and depression-like behavior (Miller and Maguire, 2014). In the hippocampus, chronic stress also causes dephosphorylation of KCC2 at S940 and the loss of KCC2 surface expression, coinciding with a depolarizing shift in E_GABA_ (MacKenzie and Maguire, 2015). In ventral tegmental area GABAergic neurons, acute restraint stress inhibits KCC2 function (Kimmey et al., 2019). Therefore, KCC2 might be downregulated in CRH neurons of pups born to food restricted mothers, which activates CRH neurons.

Exposure to prenatal and early life stress represents a major risk factor for the development of both physical and mental dysfunction later in life (Agorastos et al., 2019). Among the different consequences of exposure to stress on fetal programming, dysregulation of the HPA axis constitutes a critical node capable of affecting multiple organ systems through altered glucocorticoid signaling. Incidentally, evidence from human studies suggests that food restriction stress can trigger growth retardation (Franke et al., 2020; Gantenbein and Kanaka-Gantenbein, 2022; Mousikou et al., 2023). It is hypothesized that food restriction stress triggers multiple cascades involved in anabolic processes during fetal growth. The current consensus understanding underlying this phenomenon stems from observations of reduced nutrient transport via the placenta with key roles assigned to mechanistic target of rapamycin, adiponectin and O-GlcNac transferase signaling (Kramer et al., 2023). However, mechanistic understanding of changes in the fetus responsible for programming of future disease remain unidentified. In particular, development of the brain appears to be compromised, with findings showing reductions in cortical volume, reduced neuronal densities in limbic structures, changes in synaptic plasticity, and aberrant neurotransmission (Kassotaki et al., 2021). In cognizance of our observation of early development of GABAergic inhibitory control of CRH neuron activity, as well as significant depolarization of E_GABA_ following food restriction stress, we hypothesize that perinatal dysregulation of the fetal HPA axis through stressors may also contribute to maladaptation during this highly plastic phase of brain development. The ensuing cascade of events could be intimately linked to the etiopathology of many neurodevelopmental disorders, and therefore warrants extensive evaluation using multiple animal models of neurodevelopmental disorders.

## Data availability statement

The raw data supporting the conclusions of this article will be made available by the authors, without undue reservation.

## Ethics statement

The animal study was approved by the Animal Care and Use Committee of Hamamatsu University School of Medicine. The study was conducted in accordance with the local legislation and institutional requirements.

## Author contributions

MW: Conceptualization, Formal analysis, Funding acquisition, Investigation, Writing – original draft, Writing – review & editing. AS: Conceptualization, Formal analysis, Investigation, Writing – original draft, Writing – review & editing. YS: Conceptualization, Supervision, Writing – original draft, Writing – review & editing. AF: Conceptualization, Funding acquisition, Supervision, Writing – original draft, Writing – review & editing.
